# Dynamic bottleneck elimination in mattress manufacturing line using theory of constraints

**DOI:** 10.1186/s40064-016-2947-1

**Published:** 2016-08-08

**Authors:** Emin Gundogar, Murat Sari, Abdullah H. Kokcam

**Affiliations:** Industrial Engineering Department, Sakarya University, 54187 Sakarya, Turkey

**Keywords:** Bottleneck search, Buffer stock, Theory of constraints, Simulation, Spring mattress manufacturing

## Abstract

There is a tough competition in the furniture sector like other sectors. Along with the varying product range, production system should also be renewed on a regular basis and the production costs should be kept under control. In this study, spring mattress manufacturing line of a furniture manufacturing company is analyzed. The company wants to increase its production output with new investments. The objective is to find the bottlenecks in production line in order to balance the semi-finished material flow. These bottlenecks are investigated and several different scenarios are tested to improve the current manufacturing system. The problem with a main theme based on the elimination of the bottleneck is solved using Goldratt and Cox’s theory of constraints with a simulation based heuristic method. Near optimal alternatives are determined by system models built in Arena 13.5 simulation software. Results show that approximately 46 % capacity enhancements with 2 buffer stocks have increased average production by 88.8 %.

## Background

Each company establishes its production system (number of employees, machine capacities, buffer stocks) with the capacity to produce its product range with optimum cost. The existing production system fails to optimally respond to the product range that varies with rapidly changing fashion. Along with the modifications in the product range, the production system should also be renewed on a regular basis, keeping the production costs under control. This especially applies to the furniture sector, with its great impact on the daily lives of people. Primarily, diversities in the product range pose several impediments for product standardization in the company analyzed in this study. The company uses an overtime manufacturing policy to address this problem leading to serious increases in the operational costs, which in turn urges them to find new ways to increase the production efficiency.

A congestion, encountered in any system from computer networks to a factory assembly line, is described with the term “bottleneck”. In a system that is subject to a bottleneck, there is always a process, task, machine, etc. acting as the limiting factor for a greater throughput, thus determining the capacity of the entire system. Knowing about the bottleneck allows increasing the flow through improvement of a single process without having to intervene the whole system. In other words, the amendments made on any point other than the bottleneck, will not contribute to the output (Goldratt and Cox’s 1984).

Production lines involve various parameters such as processing times and setup times, which render keeping the balance of the line a serious challenge. As suggested by Van Delft et al. (2008) solution of such problems by means of mathematical modelling and similar deterministic methods in polynomial time is not possible due to their complexity and stochastic nature instead heuristic and approximate methods can be used for this type of problems. Goldratt and Cox’s (1992) TOC is one of the heuristic methods which can be easily implemented to a simulation environment. Therefore, the problem, main theme of which is based on the elimination of the bottlenecks, is solved using TOC by means of a simulation based heuristic method. TOC has been used in various topics such as health care, finance, production, operations management, and quality management (Mabin and Balderstone 2003; Orouji 2016). There are several theoretical works that used TOC on operations management (Boyd and Gupta 2004; Gupta and Boyd 2008), production process efficiency (Andelkovic et al. 2013), supply chain management (Puche et al. 2016).

Bottleneck elimination process has four main steps which should be investigated thoroughly. These are material, machine and man (workforce), respectively (Üstün 2005). Bottleneck detection and elimination methods have been studied since the industrial revolution, since detection of a bottleneck in a production system is a complex task. In the literature, there have been numerous studies about current bottleneck detection methods which can be divided into two categories as analytical and simulation-based (Li et al. 2007).

Analytical methods yield satisfactory and reasonable results for long term analyses but they are not suitable for short term analyses, since they can give misleading results in short time intervals. There are several works including both fictive (Balbo and Serazzi 1997; Amirghasemi and Zamani 2014) and experimental (Li et al. 2009) case studies, and different analytical methods have been applied such as queuing theory (Lawrence and Buss 1995) and Markov chain theory (Chiang et al. 2001), for elimination of bottlenecks.

Analytical approaches are not as sufficiently applicable as simulation based methods for real manufacturing processes due to highly complicated dynamic/stochastic structure of the problem. On the other hand, simulation based methods have some disadvantages such as the time required for modelling since different scenarios require different models.

Simulation based methods are widely used in many areas such as production policies (Al-Harkan and Hariga 2007), inventory model (Firoozi et al. 2013), scheduling (Baesler et al. 2015), maintenance (Razavi et al. 2015), assignment (Lam et al. 2014) problems and also used in bottleneck elimination problems as following.

Leporis and Králová (2010) applied several methods in simulation environment to detect bottlenecks in manufacturing processes and compared the advantages and constraints related to these methods. Scholz-Reiter et al. (2011) developed a systematic and comprehensive definition of dynamic bottlenecks of the production networks based on both the TOC and the bottleneck-oriented logistic analysis. Gopalakrishnan et al. (2014) investigated how a shifting priority strategy could be integrated into the scheduling of reactive maintenance. They applied the strategy in an automotive case-study, using simulation for decision support. They showed how to shift prioritization by tracking the momentary bottleneck of the system.

On the other hand, in recent years, researchers have developed new hybrid methods and techniques such as meta-heuristic algorithms with simulations (Volling and Spengler 2011).

There are several studies (Sari et al. 2013; 2014a; b) similar to this one using only capacity increase to overcome bottleneck elimination problem.

Simulation methods are widely applied to investigate and estimate bottleneck problems in a workflow. The aim of the current study is to improve the flow of products by reducing bottlenecks in production line using simulation tools. The company receives orders from a wide range of dealers and directly transports the products to sub-vendors. In the current case, the company produces an average of 200 (±5) products. The company aims to reduce the unit product costs as much as possible through maximization of its daily output with required capacity enhancements. This is a case study to increase the production, based on factual data that have been acquired from a factory by modelling discrete event simulation (DES) of the production line.

The rest of the study is organized as follows. Detailed analysis of the product and the production line is given in the second section. Work study and input analysis is given in the third section. Definition, methodology, and application areas of the “theory of constraints” are explained in fourth section. Conceptual simulation model and application of package program is given in the fifth section. Verification and validation of the system is given in sixth section. Finally, simulation results and conclusion are given in the seventh section.

## Manufacturing system description

**Fig. 1:**
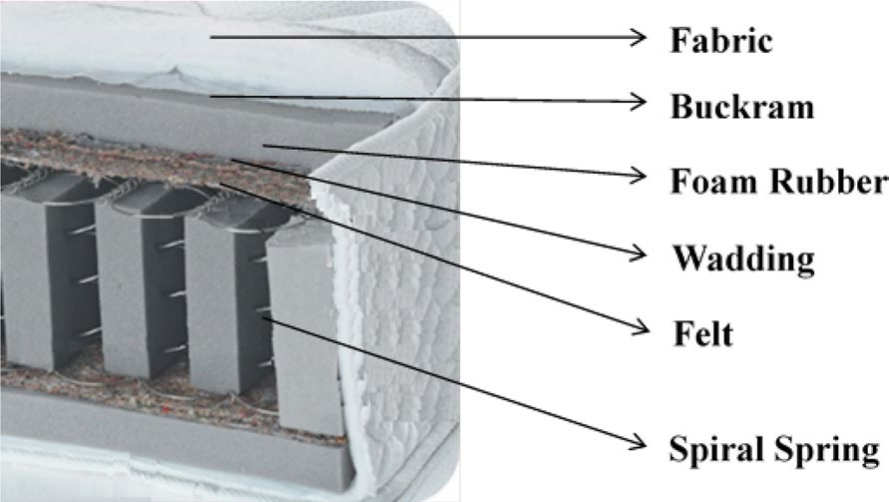
The structure of classical spring mattress

A furniture company produces different spring mattresses. To understand the problem of interest, the materials used in a classical spring mattress are summarized in Table 1 and shown in Fig. 1.

**Table 1:** Descriptions of materials used in spring mattress

Material name	Description
Fabric	A woven cloth of organic or inorganic filaments
Mattress frame	Mattresses are constructed by knitting spiral springs, which are made of high carbon reinforced steel fibre processed with heat treatment
Buckram	A coarse cloth made of linen or hemp, stiffened with size or glue, which is used in garments to keep them in the desired form
Felt	Nonwoven fabric made by stratifying thin sheets of carded wool fibers, which is processed under heat, moisture, and pressure to shrink and compress the fibers into a thick matted cloth that will not ravel or fray. The felt uniformly distributes the weight on bed surface
Foam rubber	Light and spongy rubber which is used as a padding material in the mattress
Wadding	Soft fibrous cotton or wool material which is used for stuffing (wad) between fabric and mattress frame

**Fig. 2:**
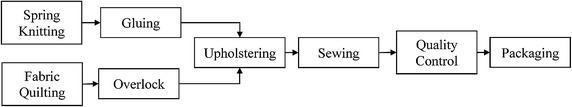
Standard mattress manufacturing process

Spring mattress manufacturing line consists of eight work stations, namely Spring Knitting, Gluing, Fabric Quilting, Overlock, Upholstering, Sewing, Quality control and Packaging. The mattress manufacturing process starts in two different lines. Mattress frame is built in the Spring Knitting station at the first line. Then appropriately sized felt and foam rubber are glued up to that mattress frame in the gluing station. The second branch of manufacturing line consists of Fabric Quilting and Overlock stations. Fabric, buckram, wadding and foam rubber are sewn with appropriate designs (pattern) according to the features of mattress being produced. Afterwards, quilted fabric is overlocked if necessary. After the completion of processes in these two lines, semi-finished products (mattress frame and quilted fabrics) are combined in Upholstering station. Top and bottom quilted fabrics and lateral fabric (called bordure) are combined with straight binding process in the sewing station. In the quality control station, scrap suture strands are removed from spring mattresses and physical examination test is carried out. Finally, the spring mattress is packaged automatically in the package station. Main steps of spring mattress manufacturing process are summarized in Fig. 2.

## Work study and input analysis

As remarked by Law and Kelton (1991), a thorough and detailed modeling of a system is time consuming and unnecessary in most cases. Indeed, as the number of the details increase, verification and validation of the model becomes more difficult, increasing the error risk. Therefore the details with no significant impact on the production system, are not included in the model. Twenty different types of mattresses are manufactured by the company, each having seven different sizes. However, five most demanded types and sizes of mattresses are specified with ABC analysis (Reid and Sanders 2012) to be used in the simulation. It should be noted that the impact of other demands is neglected. Pattern of demand is not require to change production within a day. Thus it is planned that no batch change has been made during the day. Each type of selected mattress frame is manufactured from a different type of bonnell spring. Other specifications of these mattresses are listed in Table 2.

**Table 2:** Specifications of mattresses

Type	Fabric	Comfort	Overlock	Size (cm)
A	Jacquard	Ergonomic	No	90 × 190
B	Jacquard	Ergonomic	No	70 × 180
C	Non-sweat	Ultra ergonomic	Yes	150 × 200
D	Non-sweat	Ultra ergonomic	Yes	150 × 200
E	Jacquard	Standard	No	150 × 200

Production line flow is thoroughly observed and analyzed. Setup and process times of all processes are determined using the time study and statistically evaluated using “Arena input analyzer” software (Zupick et al. 2014).

**Fig. 3:**
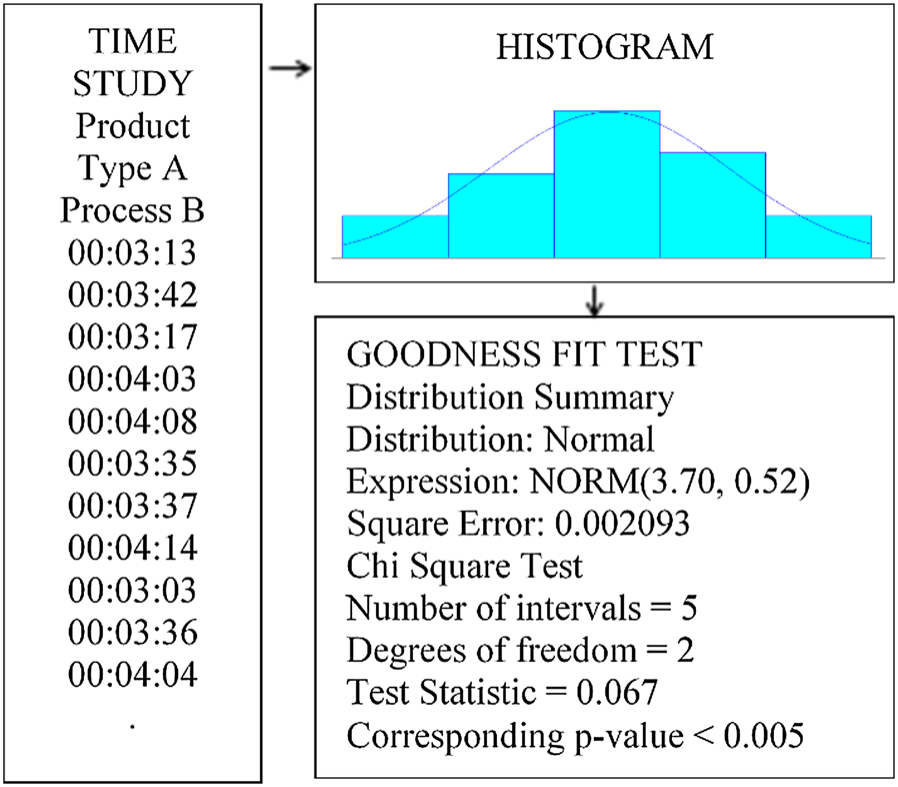
Time study

In this analysis, goodness of fit (Chi square) tests are applied. Mean square error of the tests is considered to be less than 5 %. Results of the time study and input analysis were found too long to include in this paper so an example of these analyses are shown in Fig. 3.

After the analyses, machine setup times, current capacities of stations and changeability of capacity are given in Table 3. The company management declared that the capacity of fabric quilting and packaging stations could not be modified, due to the high investment costs which are likely to add to the constraints already available in this problem. Also, the company can afford maximum two buffer stock areas.

**Table 3:** Machines’ specification

Machine codes	Machine name	Setup times (min)^a^	Current capacity	Modifiability of capacity
r1	Spring knitting	15	2	Yes
r2	Glue	15	2	Yes
r3	Fabric quilting	10	1	N/A
r4	Overlock	N/A	1	Yes
r5	Upholster	N/A	1	Yes
r6	Sewing	1.56	2	Yes
r7	Quality control	N/A	2	Yes
r8	Package	3.23	2	N/A

*N/A* not applicable

^a^Distribution of the setup times are ignored due to high production quantities

Company do not want to waste time with setup times as a production strategy. In order to do that they do not frequently change manufactured product. They start with a product and continue to produce till the order finishes. After that they set-up machines and start to produce next product.

In this scope, after the capacity enhancements completed buffer stock locations are determined, if required. Besides that determining the amount of buffer stocks are out of the scope of this study. On the other hand there are works such as Reyes et al. (2015) which have been studied the sensitivity of buffer stock amount using TOC methodology.

Processing time matrix of the stations are given in Tables 4 and 5.

**Table 4:** Processing time matrix for spring knitting, gluing, fabric quilting, and overlock stations (min)

Product name	Spring knitting	Gluing	Fabric quilting	Overlock
Type A	unif (4.07, 6.83, seed1)	norm (3.70, 0.52, seed6)	norm (0.52, 0.05, seed11)	N/A
Type B	unif (2.03, 3.26, seed2)	norm (2.04, 0.26, seed7)	norm (0.47, 0.13, seed12)	N/A
Type C	unif (4.39, 6.42, seed3)	norm (3.93, 0.93, seed8)	norm (0.71, 0.05, seed13)	unif (1.10, 1.59, seed16)
Type D	unif (4.39, 6.42, seed4)	norm (3.67, 0.55, seed9)	norm (0.71, 0.05, seed14)	unif (1.10, 1.59, seed17)
Type E	unif (4.39, 6.42,seed5)	norm (3.93, 0.93, seed10)	norm (0.63, 0.06, seed15)	N/A

*N/A* not applicable

**Table 5:** Processing time matrix for covering/upholster, sewing, quality control, and packaging stations (min)

Product name	Covering/upholster	Sewing	Quality control	Packaging
Type A	norm (2.43, 0.25, seed18)	unif (2.50, 5.00, seed23)	norm (0.82, 0.35, seed28)	0.65
Type B	norm (1.40, 0.19, seed19)	unif (1.75, 3.34, seed24)	norm (0.79, 0.35, seed29)	0.67
Type C	norm (2.34, 0.15, seed20)	unif (3.29, 5.80, seed25)	norm (1.46, 0.18, seed30)	0.78
Type D	norm (2.34, 0.15, seed21)	unif (3.29, 5.80, seed26)	norm (1.46, 0.18, seed31)	0.78
Type E	norm (2.34, 0.15, seed22)	unif (3.29, 5.80, seed27)	norm (1.46, 0.18, seed32)	0.78

## The theory of constraints

In a production or service system all processes are related with its predecessor and successor if available. Each process has a limited production capacity within its constraints. In almost all cases there is only one process that limits or restricts the performance of entire system (Chapman 2005). Theory of constraints is based on the premise that the rate of goal achievement by a goal oriented system is limited by at least one constraint and adopts the common idiom as “a chain is no stronger than its weakest link”. That is to say systems or part of systems are vulnerable because the weakest element or part can always damage or break them or at least adversely affect the output. In other words if there was no obstacle that prevents a system from achieving higher throughput, its throughput would be infinite which is not possible in a real-life system. Overall throughput can be increased only by increasing the flow through the constraint (Goldratt and Cox 1984).

Assuming the goal of a system has been articulated and its measurements defined, the logical thinking is based on a continuous improvement cycle with five steps (Goldratt and Cox 1992):

1. Identify the bottleneck,
2. Decide how to exploit the bottleneck,
3. Subordinate everything else in the system to the previous step,
4. Elevate the bottleneck, and.
5. Evaluate if the bottleneck has been broken, and return to the beginning

These five steps of TOC has been applied to find out bottlenecks and get information about elevating them from the production system.

## Simulation model

During the stage of setting a simulation model, “progressive refinement” and “increased expansion” techniques, recommended by Law and Kelton (1991), are used and their suggestions to the modeling stage are considered. According to these suggestions, although there are established rules in some of the companies for the modelling process, there is a consensus on starting with the simple model and adding on the details when required. The model should involve only sufficient amount of details. One-to-one pairing with all components of the system is not required. This way the probability for occurrence of the errors will be minimized (Law and Kelton 1991).

Firstly, orders are defined in the system. In the second step, all machinery (sources) and their capacities within the production environment are defined. In the third step, the routes between the machines are defined. In the fourth, the rules and matrixes required for integration of assets (raw material, semi products) during the production process, were established. In the last step, the animation of overall system is prepared and synchronized with the model.

Production line is left as it is after finishing the 8 h of daily work and continues from there in the next working day. So reoccurring of steady state is beside the point as long as orders of that product are not completed. In other words production line is continuously produce until the orders of a product is finished. Therefore type of this system is non-terminating.

In the simulation it was assumed that;

- The machines do not malfunction
- No preventive maintenance is carried out on the machines
- There is no defective manufacturing
- All material stocks are sufficient
- A net working day is 8 h (after subtracting the tea and lunch breaks)
- There is no physical obstruction within the production environment
- Initially, there is no work in process

**Fig. 4:**
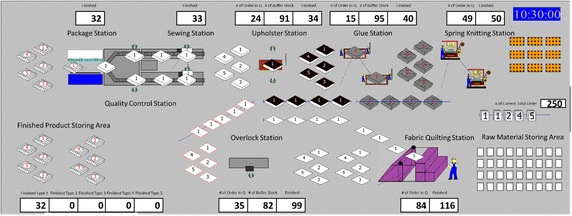
Animation interface of simulation model

The animation interface of simulation model, established as a result of implementation of all abovementioned steps in Arena Simulation Software, is given in Fig. 4 (Pegden et al. 1995; Rossetti 2015).

There are more than one way to eliminate a bottleneck. The foremost and easy to use one of these is the increasing the source capacity of a bottleneck. Besides that there are various methods that could eliminate a bottleneck by shifting that bottleneck to another process and help achieve output goals such as regulating break times, effective planning on production schedule of sources (using proper queue discipline), using buffer stocks, and improve production processes by R&D studies, etc. In this context, production quantities are increased with two steps. Capacity of sources increased in the first step and buffer stocks are used in the second step to utilize dead times when a source got starve.

**Fig. 5:**
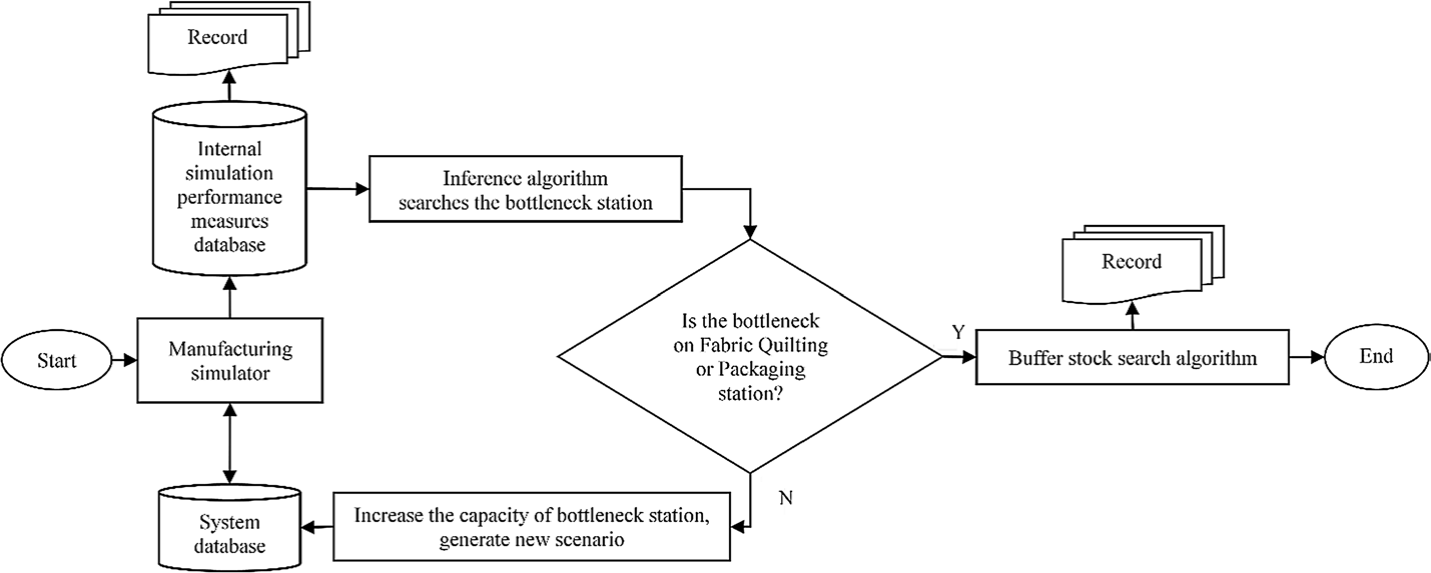
Bottleneck elimination algorithm embedded DES diagram of manufacturing model

In each production period, the manufacturing line should be re-balanced because of the modifications in the product range. Line balancing focuses on the workers and the machine capacity. In the first step, different iterative simulation scenarios are tried to increase the daily mattress production, and the second step focuses on the types and locations of buffer stocks (after the spring knitting, fabric quilting, gluing, and overlock stations) to run the manufacturing line without a hitch. Goldratt and Coxs’ (1984) bottleneck elimination algorithm embedded DES flow diagram of manufacturing model is given in Fig. 5.

This closed loop algorithm improves models in each iteration by using current simulation model. Quantity of daily production units are taken as a performance measure for algorithm. Starting with the initial model which is the current state of the firm, manufacturing simulator calculates the values and records the internal simulation performance measures database.

Algorithm records the quantity statistics of semi-finished products. In other words queue lengths of sources are calculated. According to TOC, bottleneck source has the longest queue of all sources in a production system. Thus bottleneck to work on can be identified. Simulation is re-run after making required improvements to find out the source which have the longest queue until the objective quantity achieved or a stopping criteria is met.

Inference algorithm searches for the bottleneck station from these values. There is a control stage which checks whether the bottleneck station is Fabric Quilting or Packaging stations. Algorithm increases the capacity of bottleneck station and generates a new scenario, if there are bottleneck in one of these stations. This new scenario is recorded to system database and sent to the manufacturing simulator for calculations. Algorithm try to find better scenario as long as there are bottleneck in these stations.

Otherwise, buffer stock search algorithm is executed to find appropriate buffer stocks for the stations using brute force method. It finds out all possible combinations of buffer stock locations and simulate with high quantities of buffer stocks. After that calculations are recorded and algorithm is terminated. In this case, theoretically there are 7 different buffer stock locations. Thus there are 28 $$\left( {\left( {\begin{array}{*{20}c} 7 \\ 1 \\ \end{array} } \right) + \left( {\begin{array}{*{20}c} 7 \\ 2 \\ \end{array} } \right)} \right)$$ different scenarios that can use 1 or 2 different buffer stock locations.

While evaluating the outputs of the scenarios, the point estimates for performance values (production quantities) will not be sufficient in terms of statistical evaluation. Accordingly, the decision maker will have to evaluate the confidence intervals (CI) of the performance values. A certain number of experimental results will be required for confidence interval evaluation. For this, the experiments will be replicated—with different random number series. The half-width (h) values, as a non-biased deviation measure, will be calculated for the obtained result data. The h value is calculated as in Eq. 1.

1 $$h = t_{{{{n - 1, \left( {1 - a} \right)} \mathord{\left/ {\vphantom {{n - 1, \left( {1 - a} \right)} 2}} \right. \kern-0pt} 2}}} \times \frac{s}{\sqrt n }$$

In this equation; t shows the corresponding t-distribution table value of test model, n is the sample size (number of replications), α is the significance level of the test (percent of confidence interval), and s is the standard deviation of samples (outputs).

Increasing the number of replications will also increase the accuracy, and it will reduce the half-widths of output parameters. In this study, the half-width values are calculated so that maximum one product shows deviation, using the number of replications within the equation in the literature (Firoozi et al. 2013). In this point we took the parameter based on deviation of daily production quantities. It can be seen in Tables 6 and 7 that half-width values cannot be higher than +1.00. Consequently, sufficient number of half-width values was reached when the number of replications was 34. The number of replications is calculated as shown in Eq. 2.

2 $$n^{ *} = \left[ {n \times \left( {\frac{h}{{h^{ *} }}} \right)^{2} } \right] + 1$$

**Table 6:** Average production quantities and half-width values of iterative resource capacity simulation runnings

Scenario	Resource capacity	Type A		Type B		Type C		Type D		Type E		Average*
		Average	Half-width	Average	Half-width	Average	Half-width	Average	Half-width	Average	Half-width	
Present	r1 = 2, r2 = 2, r3 = 1, r4 = 1, r5 = 1, r6 = 2, r7 = 2, r8 = 2	165.90	0.49	323.06	0.81	165.50	0.42	165.40	0.42	165.36	0.55	197.04
S_1_	r1 = 3, r2 = 2, r3 = 1, r4 = 1, r5 = 1, r6 = 2, r7 = 2, r8 = 2	185.43	0.57	325.30	0.69	166.76	0.25	167.16	0.45	191.36	0.33	207.20
S_2_	r1 = 3, r2 = 3, r3 = 1, r4 = 1, r5 = 1, r6 = 2, r7 = 2, r8 = 2	186.46	0.59	325.80	0.70	166.76	0.25	167.16	0.45	192.30	0.34	207.70
S_3_	r1 = 4, r2 = 3, r3 = 1, r4 = 1, r5 = 1, r6 = 2, r7 = 2, r8 = 2	187.46	0.54	325.80	0.70	166.76	0.25	167.16	0.45	193.60	0.41	208.16
S_4_	r1 = 3, r2 = 2, r3 = 1, r4 = 1, r5 = 2, r6 = 2, r7 = 2, r8 = 2	237.16	0.80	358.66	1.00	166.76	0.25	167.16	0.45	197.50	0.72	225.45
S_5_	r1 = 4, r2 = 3, r3 = 1, r4 = 1, r5 = 2, r6 = 3, r7 = 2, r8 = 2	331.93	0.76	490.00	1.00	166.83	0.28	167.23	0.44	297.16	0.91	290.63
S_6_	r1 = 4, r2 = 3, r3 = 1, r4 = 2, r5 = 1, r6 = 2, r7 = 2, r8 = 2	187.46	0.54	325.80	0.70	191.10	0.20	191.40	0.32	193.60	0.41	217.87
S_7_	r1 = 4, r2 = 3, r3 = 1, r4 = 2, r5 = 2, r6 = 2, r7 = 2, r8 = 2	242.40	0.96	360.46	1.00	197.90	0.55	198.03	0.65	200.10	0.76	239.78
S_8_	r1 = 4, r2 = 3, r3 = 1, r4 = 2, r5 = 2, r6 = 3, r7 = 2, r8 = 2	331.93	0.76	490.00	1.00	296.76	0.73	296.76	0.90	297.16	0.91	342.52

**Table 7:** Final step of iterative buffer stock policy simulation runnings (Average production quantities and half-width values)

Scenario	Buffer stock	Type A		Type B		Type C		Type D		Type E		Average*
		Average	Half-width	Average	Half-width	Average	Half-width	Average	Half-width	Average	Half-width	
S_9_	S_8_ + After fabric quilting and Gluing	375.53	0.95	556.80	1.00	310.63	0.83	310.16	0.93	309.66	0.90	372.56

In this equation; n* is the new number of replications to yield new required half-width (h*), n shows the fixed initial replication to determine the number of replications (10 replications in this case), h is the half-width value obtained as a result of initial replication, and h* is the maximum half-width value approved by the decision maker.

### Model verification and validation

For verification of the model, Bottom-Up Testing as one of the most fundamental methods, is used (Balci 1998; Banks 1998). In this stage it was detected whether the model functions correctly using the debugging tool of the package software and detecting the systematic errors and code errors, the bugs were corrected and all lines of the model were checked. Additionally, limits of the model were pushed by excessive values and the system was deliberately forced to produce logical errors. This way the model was verified through debugging.

There is a warm-up period because of the non-terminating systems’ nature. When this production line started to produce a different product, it took approximately 30 min to get first product. Experiments and observations shows that steady state has been reached for all products at the end of first 60 min of simulation. Consequently warm-up period has been taken as 60 min and simulation data of first 60 min is not considered in calculations to prevent objective parameters to be affected by this statistics.

According to (Conway et al. 1986) who have in-depth knowledge on simulation, adding statistical accuracy on the simulation results may be delusive, since the simulation results may hold much or less significance based on the problem type, accordingly this should be the guidance for a simulator; if you are able to see it with bare eyes, do not dwell upon it. Similarly, Yeroğlu (2001) suggested that the most important benefit of simulation is not searching for absolute answers, therefore putting great emphasis on the accuracy of the simulation results should be avoided.

The validation of the model was provided through implementation of the procedures suggested by Law and Kelton (1991). In this stage, the algorithm and the relations were studied and model animation was thoroughly observed. It was confirmed that the current case scenario is in accordance with the data received from the company. Analytical models are designed for simple fictive models and the results are compared with t test. The fictive models in this study, however, are too large and complex to be represented by an analytical method. On the other hand the scenario results produced in a fictive fashion by the algorithm, are found to be within the solution space. As a result of these analyses, the model was validated.

## Results and conclusion

Heuristic based iterative method is utilized to improve the balance and efficiency of production line through increasing the production capacities of the stations.

Evaluation of the simulation results shows that the current bottleneck in the manufacturing line is Spring Knitting station. With a one unit increase in the capacity of Spring Knitting station, the daily manufacturing output increases and the resulting bottleneck switches to the Gluing station. Fabric Quilting station is found to be the bottleneck according to the results of the eighth iteration.

An analysis of the S_8_ results indicates that some of the resources suffer from a systematical material shortage. Since the capacity of this station cannot be increased due to the company’s constraints, after this point, this issue will be addressed by deployment of buffer stock in front of the stations, instead of increasing the capacity. Heuristic based iterative algorithm detects all possible buffer stock regions and deploys sufficient amounts of semi products in these points. The algorithm is terminated as the buffer stock position/positions with the highest outputs, are detected.

S_0_ represents the current situation of spring mattress manufacturing department. S_i_ represents the i.th scenario of simulation study. At the last column of these tables, average productions are calculated through averaging five kind of spring mattress productions.

Average production output has increased from 197 (current situation) to 342 units a day (73.6 % improvement), only by enhancements in resource capacities using the last scenario (S_8_), which has the 4, 3, 1, 2, 2, 3, 2 and 2 resource capacity for Spring Knitting, Gluing, Fabric quilting, Overlock, Upholster, Sewing, Quality control and Packaging stations, respectively, as shown in Table 6. Also, in terms of various buffer stocks, final result of production quantities with half-width values are given in Table 7.

At the end of the algorithm, the ideal solution is found in scenario S_9_. According to this scenario buffer stock must be located after Fabric quilting and Gluing stations. Other scenarios are not given to present paper appropriately.

As a result, the amount of average production reached 372 units (88.8 % improvement) by increasing the capacity near 46 % with 2 buffer stocks.

**Fig. 6:**
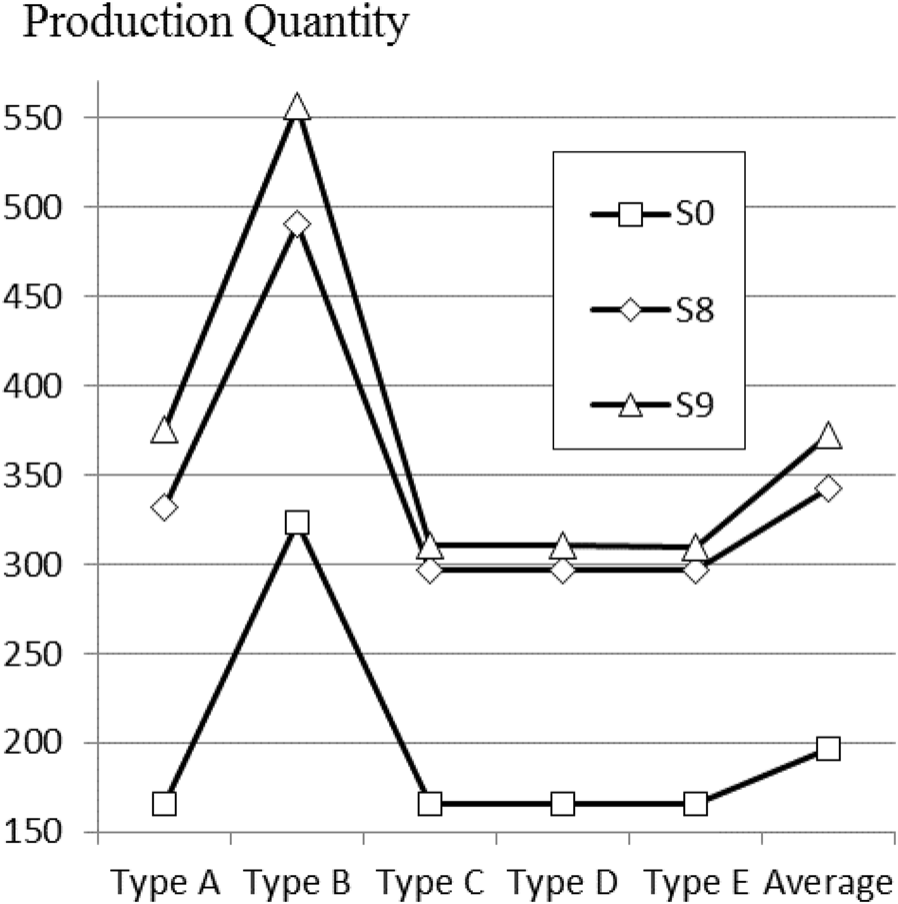
Comparison of production results of selected scenarios

The current situation, modifications on capacity suggested after the optimization and the final situation are summarized in Table 8. Also the selected scenarios and their daily production results are illustrated in Fig. 6.

**Table 8:** Summary of machine capacities

Machine codes	Machine name	Current capacity	Change	Final capacity
r1	Spring knitting	2	+2	4
r2	Gluing	2	+1	3
r3	Fabric quilting	1	N/A	1
r4	Overlock	1	+1	2
r5	Upholster	1	+1	2
r6	Sewing	2	+1	3
r7	Quality control	2	–	2
r8	Package	2	N/A	2

*N/A* not applicable

In the beginning there are no buffer stock in the factory. Locations in which buffer stocks were to be placed determined with this study. Buffer stock quantities are determined very high to prevent them to become a constraint as designed in model. According to the results, averagely 30 more products are manufactured by means of buffer stocks. This numbers are naturally fluctuate a little in each replication. In practice, the whole production line shall complete the buffer stock semi products consumed within 1 day for meeting the production targets. To meet this target it will be sufficient to make overtime work only in Spring Knitting, Gluing and Fabric quilting stations. These buffer stocks are allocated as the maximum amount of stock that a machine can process in a working day. Determining the optimum size of buffer stock is out of this study’s scope.

Importance and weight of these products are not given because of the data privacy policy of the company. In the light of this information focal point is to find the most balanced production system with the companies’ research team. Fluctuations in product demands are natural as this situation is anticipated. Philosophy of “efficiently producing the products with the help of TOC algorithm and simulation” is main theme of this study. In this context, changes in inputs will affect the results of this study, but the philosophy will always remain as it is.

Although TOC has been in literature nearly 20 years some researchers supported and some are ignore it. One of the reason is that TOC is not based on serious mathematics. Therefore TOC is not widely used in different areas as many as an “artificial neural networks” for example. How TOC can be applied a different area is showed in this study. The developed solution approach can be used in any similar production and service system.
